# Administration of trimethoprim-sulphadimidine does not improve healing of glandular gastric ulceration in horses receiving omeprazole: a randomised, blinded, clinical study

**DOI:** 10.1186/s12917-014-0180-0

**Published:** 2014-08-23

**Authors:** Ben W Sykes, Katja M Sykes, Gayle D Hallowell

**Affiliations:** 1 BW Sykes Consultancy, Upper Orara, NSW, Australia; 2 School of Veterinary Medicine and Science, University of Nottingham, Sutton Bonington, UK

**Keywords:** Horse, EGUS, Antimicrobial, Stomach, Glandular, Omeprazole

## Abstract

**Background:**

Interest in Equine Gastric Ulcer Syndrome (EGUS) has recently increased in part due to a growing awareness of the differences between squamous and glandular disease. The pathophysiology and epidemiology of squamous and glandular disease are different and recently it has been shown that the response of glandular gastric ulceration to monotherapy with omeprazole is poor. Given these differences it has been recommended that specific treatment guidelines be formulated for equine glandular disease and that adjunctive therapies be investigated. Along these lines it has been suggested that the addition of antimicrobials may enhance healing. The objective of this study was to investigate whether the addition of trimethoprim-sulphadimidine to omeprazole therapy would result in superior healing of naturally occurring equine glandular ulceration compared with omeprazole monotherapy.

**Results:**

Combination therapy of omeprazole plus trimethoprim-sulphadimidine could not be demonstrated to be superior to omeprazole monotherapy. Healing of the glandular mucosa was observed in 7/15 (47%; 95% CI 24 to 71%) and 3/13 (23%; 95% CI 7% to 50%) of horses in the TMPS and OMEP groups, respectively (OR?=?1.8; 95% CI 0.32 to 10.0; p?=?0.67). Improvement of the glandular mucosa was observed in 12/15 (80%; 95% CI 56 to 94%) and 9/13 (69%; 95% CI 42 to 89%) of horses in the TMPS and OMEP groups, respectively (OR?=?2.9; 95% CI 0.6 to 15.0; p?=?0.25).

**Conclusions:**

The results of the present study do not support the addition of trimethoprim-sulphadimidine to therapeutic protocols for equine glandular ulceration. Several limitations were present in the study and the use of antimicrobials as an adjunctive treatment warrants further investigation. However, given the potential deleterious consequences associated with the indiscriminate use of antimicrobials, the inclusion of antimicrobials in treatment regimes for EGUS is not justified until their efficacy is further validated.

## Background

The term Equine Gastric Ulcer Syndrome (EGUS) is widely used to describe gastric ulceration in the horse. However, by definition the term EGUS refers to a syndrome, within which numerous disease entities exist. Distinction between diseases of the squamous and glandular mucosa is important with each having a different proposed pathophysiology and risk factors [1]¿[4]. Furthermore, it has been reported that the presence of squamous and glandular ulceration within an individual are unrelated [5]¿[7] and the response of glandular ulceration to monotherapy with omeprazole appears inferior to that of squamous ulceration. In three recent studies only 25% of glandular ulcers healed with 28-35 days of omeprazole therapy at 4.0 mg/kg PO SID in direct contrast to a squamous healing rate of 78% [8]¿[10]. Together the results of these studies clearly demonstrate that the extrapolation of treatment recommendations from squamous ulceration directly to glandular ulceration is inappropriate and that specific guidelines are needed for the treatment of glandular gastric ulceration in the horse.

In humans, therapeutic protocols for glandular ulceration are dictated by the underlying disease process with *Helicobacter pylori-*associated ulcers treated with short duration (7 ¿ 14 days) triple therapy combining antimicrobials and acid suppression in a variety of protocols [11]. In contrast, NSAID-induced glandular ulceration is typically treated for 8-12 weeks with acid suppression therapy alone [12]. As of yet the pathogenesis of equine glandular ulceration is undetermined and, as such, the formulation of specific treatment recommendations based on the underlying disease process is difficult. There remains significant conflict in the literature as to the role of bacteria in EGUS with *Helicobacter*-like organisms identified in affected horses in some studies [13]¿[15], whilst other studies have failed to identify such organisms. [16],[17]. A recent study demonstrated that both gastric-adapted bacteria and opportunistic pathogens may play a role in squamous ulceration [18] and, although proof is lacking, it is suspected that bacteria may play a role in the either the development or perpetuation of equine glandular ulceration. In line with this, it has been suggested that antimicrobial therapy may be beneficial in treatment [19],[20].

Based on this the authors hypothesized that the combination of omeprazole at 4 mg/kg PO SID and trimethoprim-sulphadimidine (TMPS) at a dose of 30 mg/kg PO SID would improve outcome over monotherapy with omeprazole at 4 mg/kg PO SID in the treatment of equine glandular ulceration.

## Methods

### Animal ethics

The study was performed in accordance with the New South Wales Department of Primary Industries guidelines for clinical studies and the New South Wales Animal Research Act of 1985. Informed consent from the owner, or the trainer acting as an agent for the owner, was obtained at the time of enrolment to the study.

### Study design

A randomised, blinded, clinical study.

### Recruitment and examination

Thoroughbred horses from 5 different stables were examined during July 2012. Prior to examination all horses were fed their normal evening feed, but any remaining food was removed 6 ¿ 8 hours prior to endoscopy. Water was not withheld and horses were exercised normally on the morning of the examination at the trainers¿ discretion.

Horses were sedated with detomidine (10-20 ?g/kg bwt IV)^a^ and examined for the presence of gastric ulceration using a 3 meter flexible gastroscope^b^. The squamous and glandular mucosa were scored separately using a 4 point scale as described by the EGUS council [21]. Based on the results of the gastroscopic examination, horses that met the inclusion criteria were enrolled into the study. Inclusion criteria for the study were horses with grade 2 or greater ulceration of the glandular mucosa, in race training and expected to remain in work for the next 4 weeks, not receiving any other medical treatment for EGUS and otherwise considered to be free of other significant disease.

### Group allocation and blinding

Once enrolled into the study, horses were stratified by their existing trainer to reduce variability in the diet and management as these have been shown to be significant risk factors for glandular ulcer development [4]. Horses were then randomly allocated to receive either omeprazole alone (group OMEP) or omeprazole plus trimethoprim-sulphadimine (group TMPS) by pulling their names out of a hat. One investigator (KS) was responsible for randomization whilst the remaining investigators, including the principal investigator (BS) who undertook the gastroscopic examinations and scoring, remained blinded to the group allocation until scoring was completed and recorded. The trainers were not blinded to treatment group. The study protocol allowed for randomization to be broken in the event of an adverse event.

### Treatment protocols

Horses were fed and exercised at the trainers¿ discretion driven by their normal routine, housed in individual stalls, bedded on wood shavings and fed a diet typical of Australian racehorses [22]. All horses were fed twice daily, with the morning feed typically within 2 hours of completing exercise, and the afternoon feed approximately 12-14 hours before exercising. All horses received 2 grams, equivalent to 4 mg/kg for a 500 kg horse, of a commercially available, enteric coated omeprazole paste formulation^c^ PO 1 ¿ 4 hours prior to morning exercise. At the same time, horses in group TMPS also received 15 grams, equivalent to a combined trimethoprim-sulphadimine^d^ dose of 30 mg/kg for a 500 kg horse, of a commercially available oral trimethoprim-sulfadimidine paste^g^ PO. To comply with the local regulations for racing, omeprazole was not administered on the day of racing.

### Follow up endoscopy

Repeat gastroscopy, as described above, was scheduled between days 21 and 28. Some variation was allowed in the timing of the repeat gastroscopic examination to accommodate the horses¿ individual racing schedules. The squamous and glandular mucosa was scored and assessed separately for each horse. Where the starting grade was???2 for the particular mucosa, ulcer healing was defined as change to a grade of 0-1 as previously described [10]. Where a starting ulcer grade of???2 was present in the particular mucosa, horses were considered to have improved if the ulcer score for the region decreased by at least one grade. As previously described [23], where the starting score, for either the squamous or glandular mucosa, was???2 and subsequently changed to 0-1 the horse was considered to have both healed and improved for that mucosa as the definition of both improvement and healing had been met. Horses with a sub-maximal starting ulcer grade (<4) for the squamous or glandular mucosa were considered to have worsened if their ulcer grade for that particular mucosa increased by at least one grade.

### Statistical analysis

As no data was available at the time of the study on the expected degree of healing of glandular ulceration with either omeprazole alone of the combination of omeprazole plus trimethoprim-sulfadimidine a power calculation was not performed. Instead the horses were enrolled on the basis of their availability.

Data was assessed for normality using D¿Agostino and Pearson omnibus normality test. Data for age, weight and time between gastroscopic examinations in the 2 groups were normally distributed and 2-tailed unpaired Student T**-**tests were used to assess differences between the groups. All other data was not normally distributed. Genders between the two groups were compared using a two-sided Fisher¿s exact test. Baseline data for squamous and glandular ulcer scores were compared using a Mann Whitney U test. A Wilcoxon Paired Test was used to assess changes in ulcer scores within groups over time. A Mann Whitney U test was used to compare squamous and glandular ulcer scores overall and between the two groups. A Chi-squared analysis or Fisher¿s exact test (if >25% of entries had a frequency of 5 or less) were used to assess improvement or healing of ulcers.

Three commercially available statistical software packages were used^e-g^. The remaining 95% confidence intervals displayed use Jeffrey¿s intervals and were calculated using online statistical software^g^. Data is presented as mean?±?SD if normally distributed and median and inter-quartile ranges (IQR) if not normally distributed. Odds ratios and 95% confidence intervals are displayed for binomial data. Significance was determined when p?<?0.05.

## Results

### Horses

Twenty-nine horses (16 geldings and 13 females) aged from 2 to 7 years met the inclusion criteria and were randomly allocated into two groups with 15 horses in group TMPS and 14 horses in group OMEP. No adverse events were noted and blinding was maintained throughout the study with the exception of the one horse from group OMEP that was diagnosed with a lower respiratory tract infection during week 2 of the study. To ensure appropriate treatment, randomization for this horse was broken and it was subsequently excluded from data analysis leaving 13 horses in group OMEP. No other horse received antimicrobials in the study period.

Mean weight for the TMPS and OMEP treatment groups was 503?±?27 kg and 510?±?23 kg, respectively and not different between groups (p?=?0.52). There was no difference in baseline data between groups at enrolment for age (p?=?0.12), and gender (TMPS ¿ 7 females and 8 males; OMEP - 5 females and 8 males; p?=?0.66).

### Days to follow-up and number of race starts

The number of days to follow-up examination was not different between the two groups (24.5?±?2.3 and 25.1?±?1.9 in group TMPS and OMEP, respectively; p?=?0.51). There was no difference in the number of race starts between groups (median 2 (IQR: 0-2) and median 2 (IQR: 0-2) in group TMPS and OMEP, respectively; p?=?0.61).

### Ulcer scores

The entire squamous mucosa was adequately observed in all examinations. Residual fluid in the stomach prevented observation of the most ventral portion of the glandular body; however the entire pyloric antrum was visible in all examinations. There was no difference between median squamous ulcer scores when the two treatment groups were compared (TMPS ¿ 2 (IQR: 1-2) to 0 (IQR: 0-0; p?=?0.06) and OMEP ¿ 2 (IQR: 1-2) to 0 (IQR: 0-1; p?=?0.06)) over time. As the primary purpose of the study was to assess healing of glandular ulcers, no further analysis of squamous healing or improvement was performed.

**Figure 1:**
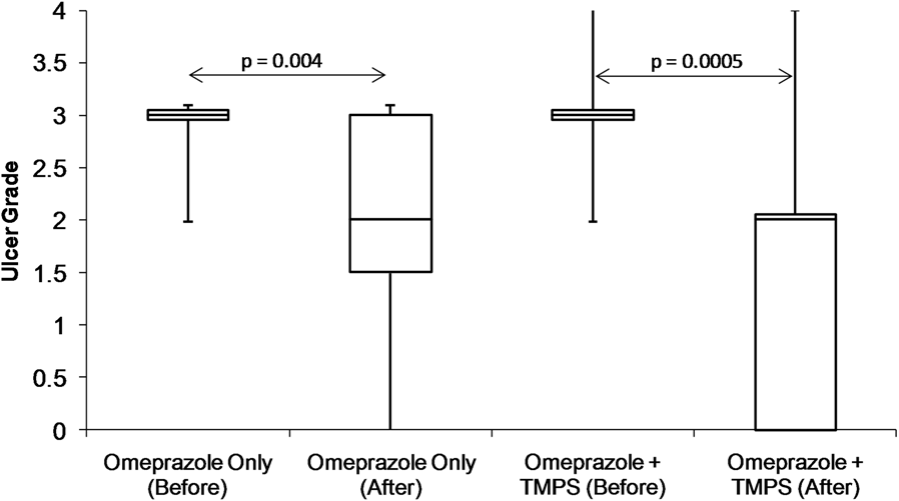
**Box and whisker plot demonstrating median (solid line), inter-quartile ranges (represented by outer edges of box) and ranges (error bars) before and after treatment for glandular ulcer grades for horses treated with omeprazole at 4 mg/kg PO SID (n?=?13) or omeprazole at 4 mg/kg PO SID plus trimethoprim-sulphadimidine (TMPS) at 30 mg/kg PO SID (n?=?15).** There was no difference between the treatment groups regarding change in glandular ulcer score over time (p?=?0.27).

There was no difference between groups regarding glandular ulcer score at enrolment (p?=?0.72). Glandular ulcer scores significantly decreased in both treatment groups (TMPS ¿ p?=?0.0005 and OMEP ¿ p?=?0.004) over time (Figure 1). There was no difference between the treatment groups regarding change in glandular ulcer score over time (p?=?0.27). Improvement of the glandular mucosa was observed in 12/15 (80%; 95% CI 56 to 94%) and 9/13 (69%; 95% CI 42 to 89%) of horses in the TMPS and OMEP groups, respectively (OR?=?1.8; 95% CI 0.32 to 10.0; p?=?0.67). Healing of the glandular mucosa was observed in 7/15 (47%; 95% CI 24 to 71%) and 3/13 (23%; 95% CI 7 to 50%) of horses in the TMPS and OMEP groups, respectively (OR?=?2.9; 95% CI 0.6 to 15.0; p?=?0.25). Worsening of glandular ulcer grade was not observed.

## Discussion

The results of the present study suggest that the addition of trimethoprim-sulphadimidine (TMPS) at 30 mg/kg PO SID does not improve the response of equine glandular ulceration compared with omeprazole monotherapy. As such the results do not support the addition of TMPS to therapeutic protocols for the treatment of glandular gastric ulceration in the horse.

Several possible reasons, beyond a true lack of efficacy, for the failure of TMPS to improve healing in this study, including the antimicrobial used, the dose given and the duration of therapy, have been identified by the authors and warrant discussion. The use of specific, targeted antimicrobial therapy is not possible in the horse due to the failure, thus far, to identify a specific pathogen in the pathogenesis of equine glandular ulceration. This is in contrast with glandular ulceration in humans where, when the organism is identified, antimicrobial selection is primarily targeted at *H. pylori*[11]. The choice of TMPS in this study was based on a its broad spectrum of activity, good penetration [24] and the high likelihood that, given its ease of use, availability and affordability, it would be selected as a first choice antimicrobial under clinical situations. Healing of squamous ulceration has been observed with 28 days of treatment of TMPS, without acid suppression therapy [18], further supporting the selection of TMPS. Whether a similar result would be observed with a different antimicrobial, or multiple concurrent antimicrobials as used in humans, is unknown but warrants investigation. However, making an evidence guided choice of such an antimicrobial is difficult and a further understanding of the role of bacteria, and the identification of specific species that may be involved in the pathogenesis of equine glandular ulceration, would be advantageous in the selection of an antimicrobial, or antimicrobials, for future trials.

An alternative explanation for the failure to see an effect of the addition of TMPS is that the dose used in the present study was too low. A range of dose recommendations, from a total daily dose of 30 - 60 mg/kg, exists for the oral administration of TMPS [25]. The dose selected in this study based primarily on four factors; firstly, it is the registered dose for the formulation used; secondly in the aforementioned study, in which squamous healing was observed with TMPS monotherapy, the total daily dose was 30 mg/kg PO [18] suggesting that an effect on gastric ulceration can be observed at the lower end of the dose range; thirdly, it has been suggested that tissue concentrations may more accurately reflect the efficacy of antimicrobials than plasma concentrations and tissue chamber fluids of both trimethoprim and sulphadiazine remain above MIC for at least 24 hours following the administration of 30 mg/kg PO in fed ponies [24]; and lastly, fasting improves the bioavailability of trimethoprim-sulphachlorpyridazine by approximately 50% [26]. Although the horses in this study were not specifically fasted, the authors have observed that the majority of animals consume their meals within 4 hours resulting in an effective fasting period of around 8 -10 hours each day prior to administration of the medications the following morning. The authors propose that it is likely that this would have resulted in greater bioavailability of TMPS potentially further enhancing tissue concentrations above that seen in fed animals. Taking the reported tissue concentrations and the possible effect of a brief fast on absorption together, the authors consider that it is likely that tissues concentrations were maintained above MIC with the dose used.

The last potential explanation for the failure of TMPS to improve healing is that the duration of therapy may have been inadequate for an effect to be seen. In humans with *H. pylori* associated ulcers, triple therapy combining antimicrobials and acid suppression consistently yields first line eradication rates of greater than 80% with 7 ¿ 14 days of therapy [11]. As such, the authors consider it unlikely that a longer duration of therapy would have resulted in a different outcome.

In the absence of an effect of the above factors the results of this study suggest that TMPS does not enhance healing of equine glandular ulceration. The first limitation of the study was the small number of animals studied and the risk of a type II error wherein an effect was present but not demonstrated should be considered when interpreting the results of the study. The raw data suggests that an effect may be present while the wide confidence intervals suggest that the point estimates of efficacy are likely to be imprecise and that the risk of a type II error is high. Considering this, the authors believe that it warrants investigation in a larger population whether a higher dose of TMPS (30 mg/kg PO BID), or the use of an alternative antimicrobial, would have resulted in superior healing. However, given the potential deleterious consequences associated with the indiscriminate use of antimicrobials, the authors argue that the widespread usage of antimicrobials in the treatment of EGUS should be discouraged until studies into the efficacy of antimicrobials document a positive effect. The second limitation of the study was that samples for bacterial culture and histopathology were not obtained from the gastric mucosa and should certainly be undertaken were this study carried out in a larger population.

## Conclusions

The results of this study do not support the addition of TMPS to therapeutic protocols for equine glandular ulceration. Whether the use of different antimicrobials, or a higher dose of TMPS, would result in enhanced healing is not clear but warrants investigation. However, based on the present study, the inclusion of antimicrobials in treatment regimes for equine glandular ulceration is not justified until their efficacy is further validated.

### Manufacturers addresses

^a^Dozadine, Axon Animal Health, Belrose, NSW, Australia; ^b^Portascope, Portascope.com, Bradenton, Florida, USA; ^c^Gastrozol, Axon Animal Health, Belrose, NSW, Australia; ^d^Ilium Sulprim, Troy Laboratories, Glendenning, NSW, Australia; ^e^GraphPad 6.0, Graphpad Software, La Jolla, California, USA; ^f^SPSS for Windows 21.0, SPSS Inc, Chicago, Illinois, USA; ^g^http://epitools.ausvet.com.au/content.php?page=CIProportion; ^h^Winpepi Compare 2 3.08. www.brixtonhealth.com/pepi4windows.html

## Competing interests

None of the author¿s received payment for performing the study, nor do any have relationships, financial or otherwise, that could reasonably be expected to influence the outcome of the study.

## Authors¿ contributions

BS designed the study, performed the gastroscopic examinations, collated the data and drafted the manuscript. KS contributed to the execution of the study. GH contributed to the study design and performed the statistical analysis. All authors have read and approved the manuscript.
